# Lipopolysaccharide-Induced Inflammatory Response and Its Prominent Suppression by *Paspalum thunbergii* Extract

**DOI:** 10.3390/ijms26041611

**Published:** 2025-02-13

**Authors:** Bin Ha, Ji-Hye Kang, Do Hyun Kim, Mi-Young Lee

**Affiliations:** 1 Department of Medical Science, College of Medical Science, Soonchunhyang University, Asan-si 31538, Chungcheongnam-do, Republic of Korea; gkqls0988@naver.com; 2 Department of Medical Biotechnology, College of Medical Science, Soonchunhyang University, Asan-si 31538, Chungcheongnam-do, Republic of Korea; addio_a@naver.com; 3 Department of Research and Development, Eshel Biopharm Co., Ltd., Asan-si 31538, Chungcheongnam-do, Republic of Korea; d6811821@gmail.com

**Keywords:** *Paspalum thunbergii*, anti-inflammatory effect, MAPK pathway, NF-κB pathway, JAK/STAT pathway, Wnt/β-catenin pathway

## Abstract

The extract of *Paspalum thunbergii*, a native perennial herb in Korea belonging to the rice family, was investigated for its anti-inflammatory activity and the underlying mechanisms driving its effects. Fifteen chemical components of the *P. thunbergii* extract, including rosmarinic acid and isoquercitrin, were identified using LC-MS. The extract showed antioxidative activity through DPPH and ABTS cation radical scavenging activity. The *P. thunbergii* extract significantly inhibited lipopolysaccharide (LPS)-induced nitric oxide (NO) production in macrophage RAW 264.7 cells. The extract inhibited the expression of lipopolysaccharide-induced iNOS and COX-2, which are inflammation-related enzymes. To explore the underlying anti-inflammatory mechanism, the expression levels of signal proteins related to MAPK, NF-κB, JAK/STAT, and Wnt/β-catenin signaling were measured. As a result, the *P. thunbergii* extract inhibited the expression of p-p38, and p-JNK increased by LPS in RAW 264.7 cells. Additionally, it decreased the expression of LPS-induced p-IKKβ and p-NF-κB p65 and prevented the migration of p-NF-κB into the nucleus caused by LPS. Notably, p-JAK1, p-STAT3, Wnt 3α, β-catenin, and p-GSK-3β protein expressions were also inhibited. Therefore, the prominent anti-inflammatory activity of the *P. thunbergii* extract may be via the MAPK, NF-κB, JAK/STAT, Wnt/β-catenin signal pathway.

## 1. Introduction

The inflammatory response is a complex defense mechanism stimulated by various external stressors, including exposure to endotoxins, such as lipopolysaccharides (LPSs) [1,2], which cause tissue damage and diverse inflammatory diseases. Various medicinal plants and phytochemicals have been identified as potential anti-inflammatory agents. The current applications of natural products from plants as alternative medicines for the treatment of diverse health disorders are rapidly increasing [3]. There are various synthetic drugs for treating inflammation, such as steroidal and nonsteroidal anti-inflammatory drugs, which may cause adverse effects [4]. Therefore, natural anti-inflammatory agents with the highest efficacy and lowest degree of unwanted side effects are needed. According to the inventory list of the WHO, there have been more than 20,000 species of medicinal plants [5]. Crude extracts, phytochemicals, and secondary metabolites of medicinal plants have been used for potential pharmaceutical applications and contributed to the development of approximately half of the current drugs [6].

LPS, commonly recognized as an endotoxin or pyrogen, serves as a distinctive chemical element of the outer membrane of Gram-negative bacteria [7]. LPS stimulates the Toll-like receptor 4 (TLR 4) of macrophages to activate the mitogen-activated protein kinase (MAPK), NF-κB, and Janus kinase/signal transducer, and transcription activator (JAK/STAT) to increase inflammatory cytokines, such as TNF-α. LPS also emits inflammatory mediators like nitric oxide (NO) and prostaglandin E2 (PGE2) to initiate inflammatory responses [8,9]. Currently, Wnt/β-catenin signaling is also induced by LPS stimulation. Effective intervention in the deregulation of the Wnt/β-catenin signaling pathway may provide therapeutic opportunities for the treatment of diverse inflammatory disorders [10].

*Paspalum* is a genus of rice, distributed in several regions, including Asia, South America, and North America [11]. Information on *Paspalum* as a staple food for humans and its nutritional value may not be well documented compared to more common grains of rice or wheat. Instead, certain species of *Paspalum* are predominantly used as forage for livestock, whereas others are viewed as agricultural or environmental weeds [12,13].

Several *Paspalum* species have been recognized for their medicinal properties. *Paspalum scrobiculatum* has been traditionally used for diabetes management, showing anti-diabetic effects in alloxan-induced diabetic rats [14]. *Paspalum conjugatum* (Carabao grass) contains phytochemicals with antimicrobial properties, demonstrating efficacy against *Staphylococcus aureus* [15]. Among *Paspalum* species, *Paspalum thunbergii* Kunth ex Steud (*P. thunbergii*) is a native perennial herb in Korea [16]. The chemical composition of *P. thunbergii* has not yet been elucidated. In addition, details about the anti-inflammatory activity of *P. thunbergii* and its underlying molecular and cellular mechanism are not accessible. Therefore, this research focused on identifying the major chemical ingredients and assessing the anti-inflammatory efficacy and mode of action of the *P. thunbergii* extract to explore its potential as a botanical anti-inflammatory agent.

## 2. Results

### 2.1. Determination of the Content of Major Chemical Components in P. thunbergii

**Figure 1:**
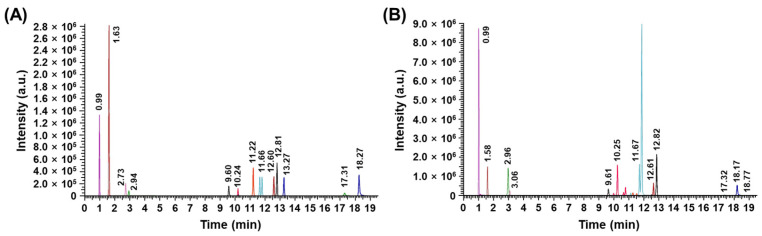
LC-MS chromatograms. (**A**) The 15 standard substances. (**B**) The *P. thunbergii* extract.

To determine the major chemical components of the *P. thunbergii* extract using liquid chromatography–mass spectrometry (LC-MS), the quantities of the compounds in the extract were quantified using the calibration data provided in Table 1. Figure 1A shows the chromatograms of the 15 standard substances measured at a concentration of 100 ppb. The evaluation of the *P. thunbergii* extract unveiled the presence of 15 compounds, classified as follows: the flavonoids class (astragalin, diosmetin, hyperoside, isoquercitrin, narcissoside, schaftoside, and vitexin), the phenylpropanoids class (rosmarinic acid, 4-O-feruloylquinic acid, danshensu), the phenolic compounds (paeonol, homogentisic acid, and eleutheroside E), the nucleosides compound (cordycepin), and other compounds (GABA) (Figure 1B). The content of these compounds in *P. thunbergii* is summarized in Table 1.

### 2.2. Antioxidative Activity of P. thunbergii Extract

**Figure 2:**
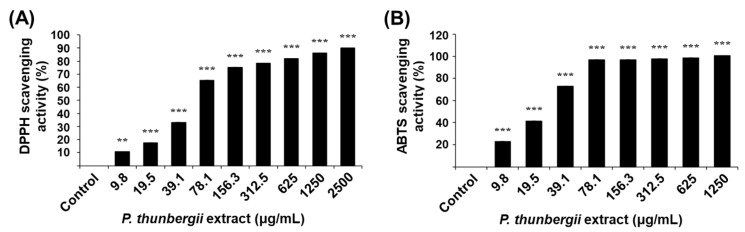
Antioxidative activity of *P. thunbergii* extract. (**A**) DPPH radical scavenging activities of *P. thunbergii* extract. (**B**) ABTS radical scavenging activities of *P. thunbergii* extract. All data are expressed as mean ± SD. ** *p* < 0.01 and *** *p* < 0.001 compared with control group. Data are taken from triplicate experiments.

To measure the antioxidative activity of the *P. thunbergii* extract, 2,2-diphenyl-1-picrylhydrazyl (DPPH) and 2,2′-azinobis-(3-ethylbenxothiazoline-6-sulfonic acid diammonium salt (ABTS) assays were performed (Figure 2). DPPH is known as a stable form of the free radical; when it is reduced by an antioxidant, its dark purple color fades to yellow [43]. When ABTS reacts with an antioxidant, cation radicals are removed and bleached from cyan to colorless [44]. In this study, the activities of scavenging DPPH and ABTS radicals increased proportionally with the concentration of the extract. These results indicate that the *P. thunbergii* extract has antioxidative activity derived from various antioxidative components capable of protecting cells from oxidative stress.

### 2.3. Inhibitory Effect of P. thunbergii Extract on LPS-Induced NO Production

**Figure 3:**
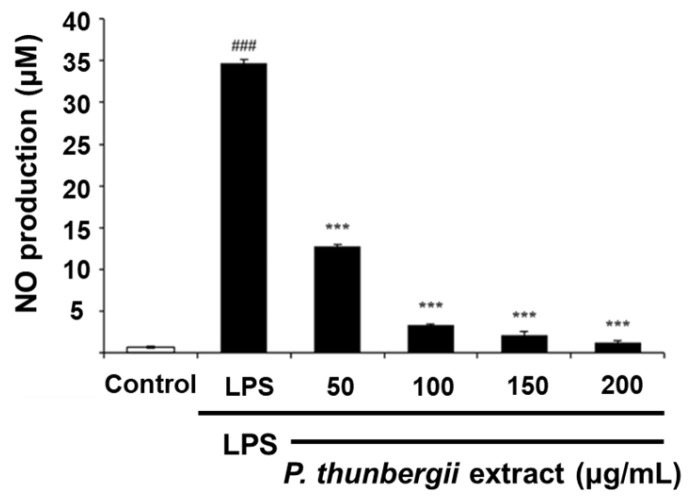
The effect of the *P. thunbergii* extract on the NO production of RAW 264.7 cells. All data are expressed as mean ± SD. ### *p* < 0.001 compared with the control group. *** *p* < 0.001 compared with the LPS group. LPS; lipopolysaccharide. Data are taken from triplicate experiments.

Cell viability in the presence of the *P. thunbergii* extract was measured using the MTT assay in RAW 264.7 cells to ascertain the extract concentration that did not induce cytotoxicity. The cells were viable with *P. thunbergii* extract concentrations ranging from 50 to 200 μg/mL. The IC_50_ value was determined to be 379.6 μg/mL. Using these concentrations, the inhibitory influence of the *P. thunbergii* extract on NO production triggered by LPS in RAW 264.7 cells was assessed using the Griess assay. The NO production amount increased to 34.7 μM with LPS treatment; however, when the *P. thunbergii* extract was treated at 50, 100, 150, and 200 μg/mL, the NO production amount was dramatically reduced to 12.8, 3.4, 2.2, and 1.2 μM, respectively (Figure 3). These findings illustrate that the *P. thunbergii* extract possesses prominent anti-inflammatory activity, primarily based on its suppression of NO production.

### 2.4. Changes in the Expression of Anti-Inflammatory Signal Proteins Through Western Blotting

**Figure 4:**
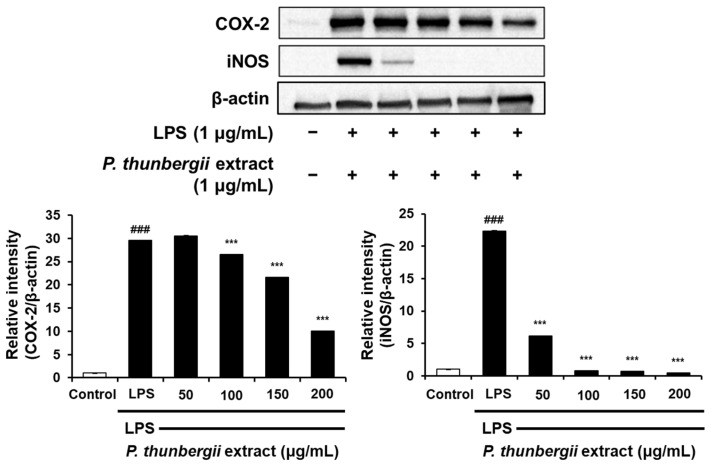
Effects of *P. thunbergii* extract on COX-2 and iNOS expression in LPS-induced RAW 264.7 cells, as shown by Western blot analysis. Expression of β-actin was used as internal control. All data are expressed as mean ± SD. ### *p* < 0.001 compared with control group. *** *p* < 0.001 compared with LPS group. LPS; liposaccharide. Data are taken from triplicate experiments.

**Figure 5:**
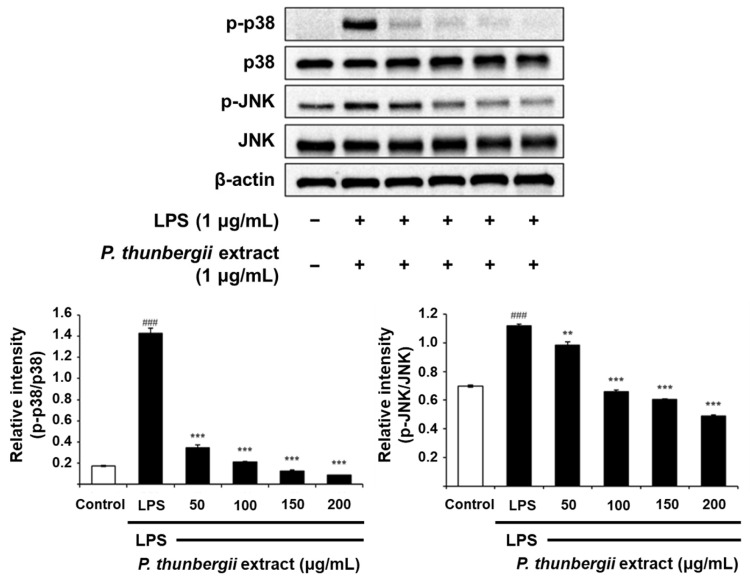
Western blot analysis of the effects of *P. thunbergii* extract on p38 and JNK signaling in LPS-induced RAW 264.7 cells. Expression of β-actin was used as internal control. All data are expressed as mean ± SD. ### *p* < 0.001 compared with control group. ** *p* < 0.01, *** *p* < 0.001 compared with LPS group. Data are taken from triplicate experiments.

**Figure 6:**
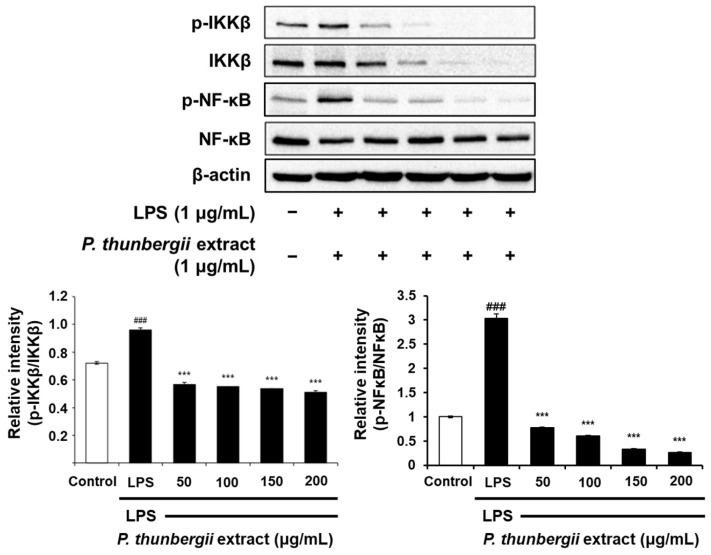
Effects of *P. thunbergii* extract on IKKβ and NF-κB expression in LPS-induced RAW 264.7 cells, as shown by Western blot analysis. Expression of β-actin was used as internal control. All data are expressed as mean ± SD. ### *p* < 0.001 compared with control group. *** *p* < 0.001 compared with LPS group. LPS; lipopolysaccharide. Data are taken from triplicate experiments.

**Figure 7:**
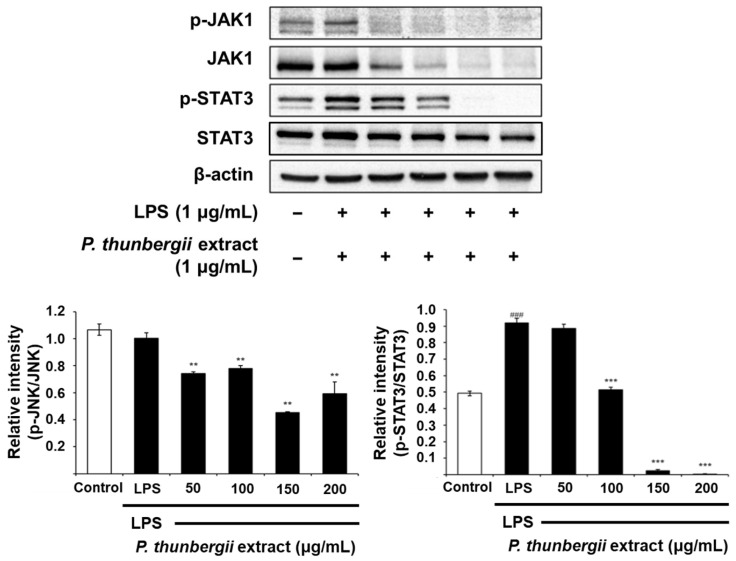
Western blot analysis of effects of *P. thunbergii* extract on JAK1 and STAT3 signaling in LPS-induced RAW 264.7 cells. Expression of β-actin was used as internal control. All data are expressed as mean ± SD. ### *p* < 0.001 compared with control group. ** *p* < 0.01, *** *p* < 0.001 compared with LPS group. LPS; lipopolysaccharide. Data are taken from triplicate experiments.

**Figure 8:**
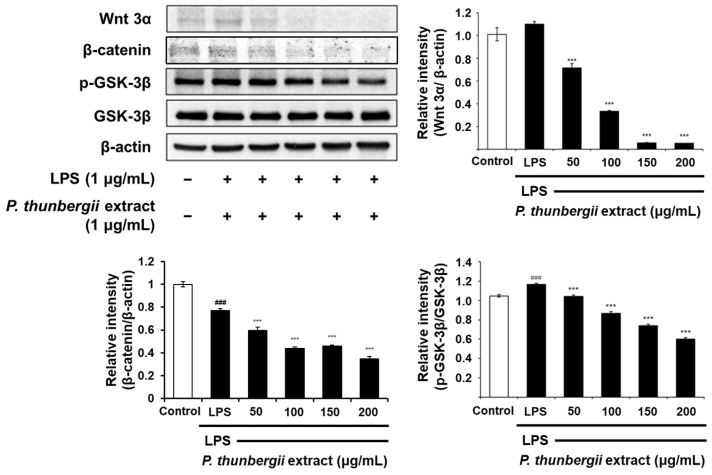
Effects of *P. thunbergii* extract on Wnt 3α, β-catenin, and GSK-3β expressions in LPS-induced RAW 264.7 cells, as shown by Western blot analysis. Expression of β-actin was used as internal control. All data are expressed as mean ± SD. ### *p* < 0.001 compared with control group. *** *p* < 0.001 compared with LPS group. Data from taken from triplicate experiments. LPS; lipopolysaccharide.

The protein-level inhibitory effect of the *P. thunbergii* extract on inflammation in RAW 264.7 cells stimulated with LPS was substantiated through Western blotting. As shown in Figure 4, the extract effectively led to a reduction in the expression of the COX-2 protein prompted by LPS. Notably, the LPS-induced iNOS expression was also dramatically reduced. Figure 5 also shows a drastic reduction in the expression of phosphorylated p38 and JNK in the MAPK pathway using the extract. In addition, the expression of the LPS-induced phosphorylated inhibitor of nuclear factor kappa-B kinase subunit beta (IKKβ) and NF-κB was downregulated upon treatment with the extract (Figure 6). Notably, the expressions of LPS-induced phosphorylated JAK1 and STAT3, which participated in the JAK/STAT pathway, were also significantly decreased (Figure 7). Moreover, the expression of Wnt 3α and β-catenin, both implicated with the signals of the Wnt/β-catenin pathway, were also markedly decreased in response to LPS (Figure 8). The amount of phosphorylated glycogen synthase kinase 3β (GSK-3β) also decreased. These results show that the *P. thunbergii* extract inhibits an inflammatory response through the obstruction of the MAPK, NF-κB, JAK/STAT, and Wnt/β-catenin pathways, thereby displaying robust anti-inflammatory activity.

### 2.5. Inhibition of Nuclear Translocation of NF-κB

**Figure 9:**
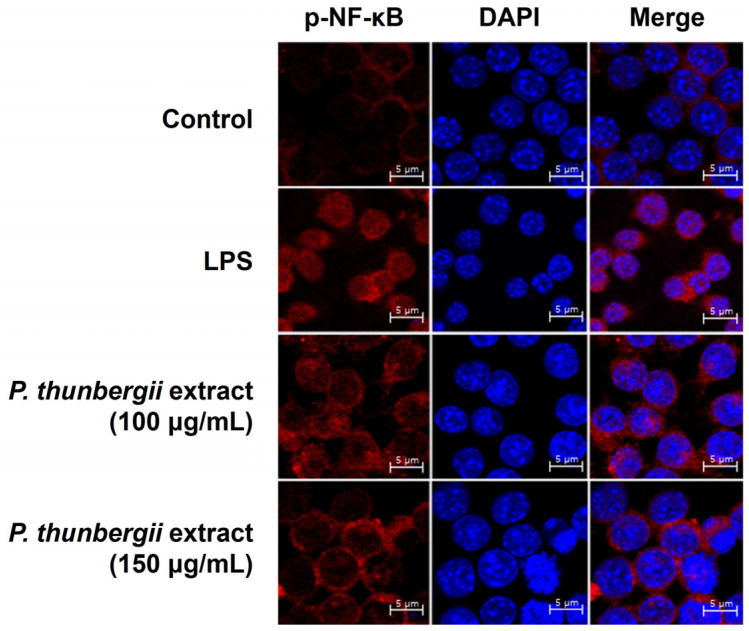
The *P. thunbergii* extract blocks the LPS-induced nuclear translocation of p-NF-κB. The expression of p-NF-κB in cells was observed using immunofluorescence. Red fluorescence indicates p-NF-κB, and blue fluorescence indicates the nucleus. Scale bar = 5 μm.

To determine whether the *P. thunbergii* extract might be involved in the inhibition of NF-κB migration to the nucleus, LPS-activated RAW 264.7 cells were treated with *P. thunbergii* extract to observe the repositioning of NF-κB using a confocal microscope. In the LPS-treated group, phosphorylated NF-κB was expressed in the nucleus; however, upon treatment with the extract, it did not move into the nucleus (Figure 9). These results show that the *P. thunbergii* extract exhibits anti-inflammation activity by obstructing the nuclear movement of NF-κB.

## 3. Discussion

The prominent anti-inflammatory activity of the *P. thunbergii* extract, and its detailed anti-inflammatory mechanisms were investigated by analyzing inflammation-related signaling pathways. NO serves as an essential inflammatory mediator that leads to the damage of cells and tissues [45,46]. NO is produced from L-arginine by iNOS, which is strongly induced by LPS and inflammatory cytokines [47,48,49,50]. In this study, the LPS-induced increase in inflammatory molecules in RAW 264.7 cells was lowered by the application of the *P. thunbergii* extract. The induction of COX-2 facilitates inflammatory reactions and is heightened during oxidative stress. In the present study, the expressions of iNOS and COX-2 increased by LPS in RAW 264.7 cells and were markedly reduced by treatment with the *P. thunbergii* extract. Therefore, this extract may have marked anti-inflammatory activity. In addition, the extract of *P. thunbergii* also exhibited antioxidative activity in the DPPH and ABTS radical scavenging assay.

NF-κB is a major signaling pathway that stimulates the expression of inflammatory mediators, namely iNOS, COX-2, and inflammatory cytokines, TNF-α and IL-6 [51,52]. The inhibitory kappa B kinase β (IKKβ) protein is phosphorylated, ubiquitinated, and degraded, inducing the activation of NF-κB and causing the transcription of inflammation-related factors [53]. Activated NF-κB is transported to the nucleus, subsequently leading to the promotion of inflammatory gene transcription [54,55]. In this study, the *P. thunbergii* extract reduced the LPS-induced phosphorylation of IKKβ and significantly obstructed NF-κB activation, thereby preventing the nuclear movement of phosphorylated NF-κB.

MAPKs, including ERK, JNK, and p38, and JAK/STAT and Wnt/β-catenin, participate in the regulation of inflammation-related gene expression, leading to the overproduction of pro-inflammatory cytokines [56,57,58,59]. The *P. thunbergii* extract inhibited the phosphorylation of p38 and JNK triggered by LPS. In addition, the extract inhibited the LPS-induced phosphorylation of JAK1 and STAT3. It also suppressed the expression of Wnt 3α and β-catenin and the phosphorylation of GSK-3β. The JAK/STAT pathway is instrumental in controlling responses to inflammation and stresses [60]. Thus, JAK/STAT signaling has emerged as a promising therapeutic target in the treatment of inflammatory and autoimmune diseases. The JAK/STAT signaling pathway comprises ligand–receptor complexes, JAKs, and STATs. There are four subtypes of the JAK family: non-receptor tyrosine kinases, such as JAK1, JAK2, JAK3, and TYK2. The selective targeting of JAK1 is a cutting-edge and effective therapeutic strategy applicable to inflammatory bowel disease treatment. Other JAK inhibitors are used to manage chronic inflammatory diseases. There are seven STAT family subtypes: STAT1, STAT2, STAT3, STAT4, STAT5a, STAT5b, and STAT6. STAT3 mediates neutrophil-driven inflammatory responses by regulating the Th17 lineage [61]. Moreover, novel anti-cancer agents target STAT3, indicating their potent clinical benefits in anti-cancer therapy [62].

The involvement of the Wnt/β-catenin pathway can be found in a wide array of biological processes, encompassing inflammation, proliferation, and cancer advancement [63]. Wnt/β-catenin signaling, identified as canonical Wnt signaling, is principally activated by adjusting β-catenin accumulation within the cytoplasm. β-catenin is recognized as a main transcriptional activator involved in the Wnt/β-catenin pathway and tightly regulated by the β-catenin destruction complex containing GSK-3β, adenomatous polyposis coli (APC), casein kinase 1 (CK1), and the axis inhibition protein (AXIN). GSK-3β, classified as a monomeric serine/threonine kinase, is involved in inflammatory regulation at various stages [64]. The activity of GSK-3 is reduced through the phosphorylation of serine 21 in GSK-3α as well as serine 9 in GSK-3β. In the present study, the *P. thunbergii* extract suppressed the LPS-induced Wnt/β-catenin pathway, causing a substantial reduction in LPS-induced Wnt and β-catenin expression. Moreover, treatment with the *P. thunbergii* extract led to a reduction in the LPS-stimulated phosphorylation of GSK-3β at serine 9, while the protein level of GSK-3β remained constant. Therefore, it is likely that the *P. thunbergii* extract exhibits an anti-inflammatory effect through the regulation of NF-κB, MAPK, JAK/STAT, as well as Wnt/β-catenin signaling pathways.

Reactive oxygen species (ROS) play a major role in many inflammatory responses, leading to a number of pathological situations [65]. Naturally occurring and synthetic antioxidants have been utilized as a protective strategy against inflammatory diseases by mitigating ROS-triggered oxidative stresses. Natural products offer an extensive array of chemodiversity that could be effectively utilized for the discovery of new therapeutic agents [66].

Therefore, the *P. thunbergii* extract displaying antioxidative activity along with an anti-inflammatory property holds the potential for creating more effective medications for inflammation treatment. In addition, the remarkable anti-inflammatory effects linked to the antioxidative activity of *P. thunbergii* may stem from the synergistic interactions of its diverse components, including the 15 identified compounds in the extract, the majority of which demonstrate both anti-inflammatory and antioxidant properties. The synergistic activity of various components in these extracts may be higher than single components [67]. Rosemarinic acid, which accounts for the largest proportion of the extract, has been shown to exhibit anti-inflammatory activity through both In vitro and In vivo studies across various inflammatory disease models, including atopic dermatitis and arthritis [68]. In addition, a substantial body of In vitro and In vivo studies related to isoquercitrin, which is abundant in the extract, highlights its potential properties across various roles, particularly as an anti-inflammatory agent, anticarcinogen, anti-diabetic compound, and anti-allergic agent [22]. In addition, standardizing the plant raw material is essential to ensure consistent efficacy and minimize variations in chemical composition due to growth conditions, facilitating its use as a reliable pharmaceutical ingredient. Overall, the *P. thunbergii* extract may represent a valuable resource in the development of anti-inflammatory therapeutics to treat inflammatory disorders induced by LPS from Gram-negative bacteria.

## 4. Materials and Methods

### 4.1. Preparation of P. thunbergii Extract

The *P. thunbergii* Kunth ex Steud. (Whole Plant) extract (KPM010-087) was provided by the Korea Plant Extract Bank of the Korea Institute of Biotechnology (Daejeon, KR). The plant was collected from Sinhyo-ro, Seogwipo-si, Jeju-do, KR in 2001. The plant (114 g), which was dried and powdered, was placed in 99.9% (HPLC class) methylalcohol and extracted using an ultrasonic extractor (SDN-900H, SD-Ultrasonic Co., Ltd., Seoul, KR) at room temperature for 30 cycles (40 KHz, 1500 W, 120 min stationary per 15 min ultrasound-1 cycle). Following filtration using qualitative filter No. 100 from Hyundai Micro Co., Ltd., Seoul, KR, and subsequent drying under reduced pressure, a total of 6.4 g of the *P. thunbergii* extract was obtained.

### 4.2. Analysis of P. thunbergii Components Using LC-MS/MS

The components of the extract from *P. thunbergii* were analyzed using LC-MS (Thermo Scientific TSQ ALTIS™, Waltham, MA, USA). The column for the separation was Kinetex Polar C18 (2.1 × 150 mm, 2.6 μm) purchased from Phenomenex, Torrance, CA, USA. Mobile phase A, which contained 0.1% formic acid in water, and solvent B, containing 0.1% formic acid in acetonitrile, were both used at a flow rate of 0.3 mL/min in a column oven set to 35 °C. The injection volume for all samples was 2 μL. A mixture of solutions containing the 15 standard compounds was prepared in 20% methanol at concentrations ranging from 1 to 200 ppb. Each solution was filtered through a 0.2 μm syringe filter. LC-MS was performed to confirm the parameters for each compound, including retention times, polarity modes (positive or negative ionization), precursor ions ([M + H]^+^ or [M − H]^−^), and product ions (*m*/*z*). The quantification and qualification of ions for each compound were selected based on the product ion scans. The ion with the highest intensity was selected as the quantification ion to ensure optimal sensitivity for precise quantification. The product ion exhibiting the second-highest intensity was designated as the qualification ion. Calibration curves were established by analyzing standard solutions at specified concentrations. The concentration of each compound in the extracts was expressed as milligrams per kilograms of the dried *P. thunbergii* extract sample (Table 2).

### 4.3. Cell Culture

RAW 264.7 cells, a murine cell line, was purchased from the Korean Cell Line Bank (Seoul, KR). RAW 264.7 cells were grown in a CO_2_ incubator at 37 °C with 5% CO_2_ in DMEM with 10% (*v*/*v*) FBS and 100 U/mL of PS.

### 4.4. DPPH Radical Scavenging Activity

To measure the DPPH radical scavenging activity of the *P. thunbergii* extract, 0.2 M of DPPH in ethanol was diluted to an absorbance of 0.3 at 517 nm. The 50 μL extract was combined with 50 μL of the DPPH reagent, and the mixture was allowed to react at room temperature for 10 min. The absorbance was recorded at a wavelength of 517 nm.

The percentage of radical scavenging activity was calculated using the following formula: DPPH radical scavenging activity % = {(A_0_ − A_1_)/A_0_} × 100, where A_0_ is the absorbance of the reagent blank and A_1_ is the absorbance of the tested sample.

### 4.5. ABTS Radical Scavenging Activity

To measure the ABTS radical scavenging activity of *P. thunbergii* extract, ABTS was dissolved in 2.5 mM of potassium persulfate to prepare a 7 mM ABTS solution and was then diluted with ethanol to achieve an absorbance value of 0.3 at 620 nm. The *P. thunbergii* extract was reacted with 50 μL of the ABTS reagent at room temperature for 10 min, and then the absorbance was assessed at a wavelength of 620 nm. The percentage of radical scavenging activity was calculated using the following formula: ABTS radical scavenging activity % = {(A_B_ − A_S_)/A_B_} × 100. Here, A_B_ is the blank absorbance, whereas A_s_ is the absorbance of the tested sample.

### 4.6. Cytotoxicity

The MTT assay was conducted to assess the cytotoxicity of the *P. thunbergii* extract. RAW 264.7 cells (3 × 10^4^ cells/well) were seeded in a 96-well plate and cultured for 24 h. Subsequently, the cells were treated with the *P. thunbergii* extract (0–400 μg/mL) for an additional 24 h at 37 °C. After incubation, the medium was discarded, and 5 mg/mL of the MTT solution was added. The cells were incubated for 2 h at 37 °C in the dark. The formazan crystals formed were dissolved in 50 μL of DMSO, and the absorbance was measured at 570 nm.

### 4.7. NO Assay

To measure the amount of NO, RAW 264.7 cells were cultured in a six-well plate at 3 × 10^6^ cells/well for 24 h. The cultivated cells were exposed to 1 μg/mL of LPS at 5% CO_2_ under dark conditions at 37 °C or treated with the *P. thunbergii* extract at various concentrations for 18 h. Each supernatant was mixed with 100 μL of the Griess agent (0.2% naphthylenediamine dihydrochloride and 0.2% sulfanilamide in 10% H_3_PO_4_), and then the absorbance was measured at 570 nm. The nitrite concentration was calculated from a nitrite standard curve, Y = 0.0086X − 0.0007, with concentrations ranging from 2.5 µM to 80 µM (R^2^ = 1.000).

### 4.8. Confocal Microscopic Analysis

After culturing, RAW 264.7 cells were seeded onto a circular cover glass coated with poly-L-lysine for 24 h. The *P. thunbergii* extract in a 1% PS-containing medium was added to the cells and cultured in the dark for 6 h. In the control group, only LPS and the medium were added, and the cells were cultured for 6 h. The cells were fixed in 1× PBS for 20 min in a 4% formalin solution and subsequently destabilized with 0.1% Triton X-100 for 15 min. After conducting the reaction using the blocking buffer, 5% BSA (Sigma Aldrich, St. Louis, MO, USA) in DPBS diluted to 1× (Welgene, Gyeongsangbuk-do, KR) for 1 h, the cells were incubated overnight with primary antibodies (with the rabbit monoclonal antibody targeting p-NF-κB p65) (1:200) (Cell Signaling Technology, Danvers, MA, USA). The cells were washed with 1× PBS for 5 min and stained with goat anti-rabbit IgG Texas Red (1:500) (Santa Cruz Biotechnology, Dallas, TX, USA) for 3 h. A circular cover slide with cells attached to a mounting agent containing DAPI (Invitrogen, Carlsbad, CA, USA) was placed on a glass slide, stained, fixed, and photographed using a confocal microscope (LSM710; Carl Zeiss, Oberkochen, Germany).

### 4.9. Sodium Dodecyl Sulfate–Polyacrylamide Gel Electrophoresis (SDS-PAGE) and Western Blotting

SDS-PAGE was performed after protein quantification in each sample. The SDS-PAGE sample buffer [250 mM of Tris-HCl (pH 6.8), 50% glycerol, 10% SDS, 0.25% bromophenol blue, and 0.5 M of DTT] and each sample was well mixed. The protein was denatured in boiling water for 3 min. The protein (20 μg) was loaded onto a flat gel with 5% acrylamide stacking and 10% acrylamide running gel. Tris-HCl (25 mM, pH 8.3) containing 192 mM glycine and 10% (*w*/*v*) SDS was used as the developing buffer at 80 volts. The proteins were segregated based on their molecular weights and subsequently transferred to a polyvinylidene fluoride membrane (Bio-Rad, Hercules, CA, USA) for 1 h in a transfer buffer (192 mM of glycine, 25 mM of Tris, and 20% methanol). After protein transfer, the membrane was placed in a 5% BSA solution and reacted for 2 h at room temperature, followed by a reaction with the primary antibodies [COX-2, iNOS, p-p38, p38, p-JNK, JNK, p-IKKβ, IKKβ, p-NF-κB p65, NF-κB p65, p-JAK1, JAK1, p-STAT3, STAT3, p-GSK-3β, GSK-3β, and β-catenin (Cell Signaling Technology), Wnt 3α, (Abcam, Cambridge, UK), and β-actin, (Santa Cruz Biotechnology)] for 18 h at 4 °C. After washing four times with a 1× TBST buffer solution, the reaction was performed using the horseradish peroxidase-conjugated anti-mouse rabbit antibody (Cell Signaling Technology) at room temperature for 1 h. The membrane washed four times, each for 10 min, in 1× TBST buffer solution, after which it was treated with ECL solution (Westar Superernova, Cyanagen, Bologna, Italy) and then photographed using an ChemiDoc image analyzer (Bio-Rad, Hercules, CA, USA) to detect the signal.

### 4.10. Statistical Analysis

SPSS (version 20, SPSS Inc., Chicago, IL, USA) was utilized for the statistical analysis of all the data and expressed as the average ± SD of the values of each group. For the comparison of each concentration, analysis was conducted using one-way ANOVA and Scheffe’s test. Statistical significance was set at *p* < 0.05. All experiments were performed in triplicate.

## 5. Conclusions

The *P. thunbergii* extract demonstrates superior anti-inflammatory effects through the modulation of the NF-κB, MAPK, JAK/STAT, and Wnt/β-catenin signaling pathways. The remarkable anti-inflammatory effects of the *P. thunbergii* extract may arise from the synergistic actions of its diverse components, which include 15 identified anti-inflammatory and antioxidative compounds. These findings indicate that the *P. thunbergii* extract possesses significant therapeutic potential as a botanical anti-inflammatory agent.

## Figures and Tables

**Table 1:** LC-MS analysis of *P. thunbergii* extract to identify main compounds, content, and therapeutic effects.

No	Compound	Structure	Content (mg/kg)	Therapeutic Effect	Refs
1	Rosmarinic acid	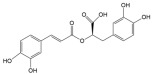	21,627.92	Anti-inflammatory Antioxidant Anti-viral and antibacterial Anti-allergic activities	[17,18,19,20]
2	Isoquercitrin	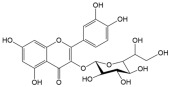	3813.47	Anti-inflammatory Antioxidant Anti-diabetic	[21,22]
3	Danshensu	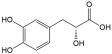	1905.95	Anti-inflammatory Antioxidant Cardiovascular protection	[23,24]
4	Schaftoside	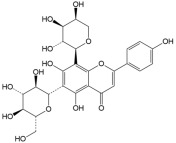	1579.8	Anti-inflammatory	[25]
5	GABA	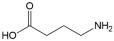	1265.05	Anti-stress Anti-anxiety Antidepressant	[26,27]
6	Hyperoside	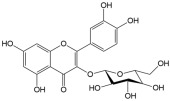	482.56	Anti-inflammatory Antioxidant Anti-cancer Anti-arthritic	[28,29]
7	Astragalin	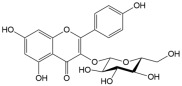	381.81	Anti-inflammatory Anti-cancer Anti-obesity Neuroprotection Anti-diabetic	[30]
8	Narcissoside	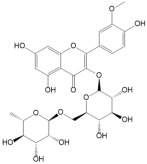	203.66	Antioxidant Anticholinergic Anti-diabetic Anti-acute myeloid leukemia	[31]
9	4-O-feruloylqunic acid	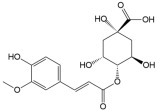	200.25	Anti-inflammatory Antioxidant	[32]
10	Diosmetin	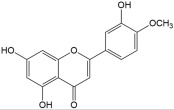	137.19	Anti-inflammatory Antioxidant Cardiovascular protection Anti-cancer	[33,34]
11	Cordycepin	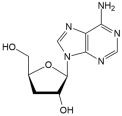	55.74	Anti-inflammatory Anti-viral Immunomodulatory Anti-cancer	[35,36]
12	Paeonol	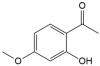	51.24	Anti-inflammatory Cardioprotective Wound healing	[37,38]
13	Vitexin	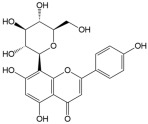	32.65	Anti-inflammatory Antioxidant Neuroprotective Anti-cancer	[39]
14	Eleutheroside E	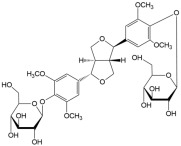	13.71	Antioxidant Anti-inflammatory	[40,41]
15	Homogentisic acid	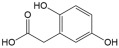	0.9	Antioxidant	[42]

**Table 2:** LC-MS analysis parameters for 15 standard substances.

No	Compound	Retention Time (min)	Polarity	Precursor Ion (*m/z*)	Product Ion *(m/z)*	Calibration Curve
1	Rosmarinic acid	13.27	Negative	359.06	161.14 132.98	R^2^ = 0.9952 Y = 4.987 × 10^3^X + 6.367 × 10^3^
2	Isoquercitrin	11.81	Negative	463.15	299.91 301.13	R^2^ = 0.9990 Y = 6.193 × 10^3^X − 6.808 × 10^2^
3	Danshensu	2.94	Negative	197.03	135.13 123.00	R^2^ = 0.9985 Y = 2.018 × 10^3^X − 1.158 × 10^3^
4	Schaftoside	10.21	Negative	562.92	353.05 443.13	R^2^ = 0.9990 Y = 2.147 × 10^3^X − 1.625 × 10^2^
5	GABA	0.99	Positive	104.08	87.13 68.99	R^2^ = 0.9994 Y = 2.515 × 10^3^X − 5.526 × 10^3^
6	Hyperoside	11.65	Negative	463.16	300.07 255.07	R^2^ = 0.9985 Y = 7.331 × 10^3^X − 1.572 × 10^3^
7	Astragalin	12.8	Negative	447.14	284.13 255.05	R^2^ = 0.9990 Y = 9.118 × 10^3^X − 1.987 × 10^3^
8	Narcissoside	12.6	Positive	625.26	317.20 479.21	R^2^ = 0.9987 Y = 8.065 × 10^3^X − 1.881 × 10^3^
9	4-O-ferulroylqunic acid	9.6	Negative	367	173.20 193.04	R^2^ = 0.9992 Y = 5.726 × 10^3^X − 1.111 × 10^3^
10	Diosmetin	18.27	Positive	301.12	286.05 258.07	R^2^ = 0.9985 Y = 9.457 × 10^3^X − 1.303 × 10^3^
11	Cordycepin	1.63	Positive	252.03	136.04 119.04	R^2^ = 0.9973 Y = 6.09 × 10^4^X + 1.636 × 10^4^
12	Paeonol	17.31	Positive	167.1	121.20 78.10	R^2^ = 0.9960 Y = 2.618 × 10^3^X + 7.744 × 10^4^
13	Vitexin	11.22	Negative	431.03	311.07 283.13	R^2^ = 0.9991 Y = 1.086 × 10^3^X − 2.636 × 10^3^
14	Eleutheroside E	10.24	Negative	741.18	417.28 579.25	R^2^ = 0.9989 Y = 4.539 × 10^2^X − 1.013 × 10^2^
15	Homogentisic acid	2.73	Negative	167.04	123.05 108.07	R^2^ = 0.9991 Y = 6.324 × 10^3^X + 8.137 × 10^2^

## Data Availability

Dataset available on request from the authors.

## Abbreviations

The following abbreviations are used in this manuscript:

ABTS	2,2′-azinobis-(3-ethylbenxothiazoline-6-sulfonic acid) diammonium salt
DPPH	2,2-diphenyl-1-picrylhydrazyl
LC-MS	Liquid chromatography–mass spectrometry
LPS	Lipopolysaccharide
NO	Nitric oxide
